# TNF Neutralization Results in the Delay of Transplantable Tumor Growth and Reduced MDSC Accumulation

**DOI:** 10.3389/fimmu.2016.00147

**Published:** 2016-04-19

**Authors:** Kamar-Sulu N. Atretkhany, Maxim A. Nosenko, Violetta S. Gogoleva, Ruslan V. Zvartsev, Zhihai Qin, Sergei A. Nedospasov, Marina S. Drutskaya

**Affiliations:** ^1^Engelhardt Institute of Molecular Biology, Russian Academy of Sciences, Moscow, Russia; ^2^Immunology Department, Faculty of Biology, Beloszersky Institue of Physico-Chemical Biology, Lomonosov Moscow State University, Moscow, Russia; ^3^German Rheumatology Research Center (DRFZ), Berlin, Germany; ^4^Institute of Biophysics, Chinese Academy of Sciences, Beijing, China

**Keywords:** MDSC, transplantable tumor model, anti-cytokine therapy, IL-6, TNF, pro-inflammatory cytokines, MCA 205 fibrosarcoma

## Abstract

Myeloid-derived suppressor cells (MDSCs) represent a heterogeneous population of immature myeloid cells (IMCs) that, under normal conditions, may differentiate into mature macrophages, granulocytes, and dendritic cells. However, under pathological conditions associated with inflammation, cancer, or infection, such differentiation is inhibited leading to IMC expansion. Under the influence of inflammatory cytokines, these cells become MDSCs, acquire immunosuppressive phenotype, and accumulate in the affected tissue, as well as in the periphery. Immune suppressive activity of MDSCs is partly due to upregulation of arginase 1, inducible nitric oxide synthase, and anti-inflammatory cytokines, such as IL-10 and TGF-β. These suppressive factors can enhance tumor growth by repressing T-cell-mediated anti-tumor responses. TNF is a critical factor for the induction, expansion, and suppressive activity of MDSCs. In this study, we evaluated the effects of systemic TNF ablation on tumor-induced expansion of MDSCs *in vivo* using TNF humanized (hTNF KI) mice. Both etanercept and infliximab treatments resulted in a delayed growth of MCA 205 fibrosarcoma in hTNF KI mice, significantly reduced tumor volume, and also resulted in less accumulated MDSCs in the blood 3 weeks after tumor cell inoculation. Thus, our study uncovers anti-tumor effects of systemic TNF ablation *in vivo*.

## Introduction

Myeloid-derived suppressor cells (MDSCs) constitute a heterogeneous population of immature myeloid cells (IMCs) that instead of undergoing terminal differentiation start to expand under the influence of inflammation, cancer, or infection (1). These cells are characterized by co-expression of CD11b and Gr-1 and can be subdivided into two populations: granulocytic CD11b^+^Ly6G^+^Ly6C^low^ and monocytic CD11b^+^Ly6G^−^Ly6C^hi^ cells (2). Initially, their accumulation was detected in cancer patients, but later an expansion of such heterogeneous population of myeloid cells with suppressive functions was also reported in several experimental carcinogenesis models in mice (3–8). It is believed that tumor microenvironment releases various factors, such as GM-CSF, M-CSF, and VEGF, which may stimulate myelopoiesis, as well as the production of pro-inflammatory cytokines, such as IL-6, TNF, and IL-1β, and anti-microbial peptides S100A8 and S100A9 (9). These factors may promote inflammation and induce activation and expansion of MDSCs *via* transcription programs controlled by STAT1, STAT3, and NFκB transcription factors (10–12). Suppressive activity of MDSCs is associated with upregulation of arginase 1 (Arg1), inducible nitric oxide synthase (iNOS), reactive oxygen species (ROS), and anti-inflammatory cytokines, such as IL-10 and TGF-β (9, 13–15). These suppressive factors can enhance tumor growth by repressing T-cell proliferation as well as T-cell- and NK-cell-mediated anti-tumor responses (16–19). Furthermore, IL-10 and TGF-β production by MDSCs may lead to the induction of T-regulatory cells and M2 macrophages with suppressive capacity (20–22). Additionally, MDSCs can attract other myeloid cells, such as neutrophils and macrophages, which further contribute to the inflammatory processes in tumor microenvironment (23).

TNF is a multifunctional cytokine involved in host defense, immune regulation, cell survival, lymphoid tissue organogenesis, and inflammation (24). TNF was initially described due to its potent anti-tumor effects against Meth A sarcoma and other transplantable tumors in mice (25). However, cancer therapy with systemically administered recombinant human TNF is associated with severe side effects due to TNF-mediated inflammation and toxicity (26). Ironically, given its name – tumor necrosis factor, it was later found that TNF may play a pro-tumorigenic role by enhancing chronic inflammation (27–30). These opposing functions of TNF in experimental carcinogenesis models can be attributed to complex signaling networks involving a constellation of TNF-producing cells and two different TNF receptors. TNF exists both in soluble and membrane-bound forms. In addition, a soluble form of lymphotoxin (sLTa and LTα_3_), a molecule closely related to TNF, can also signal through the same receptors (31). It was recently demonstrated in a transplantable tumor model that TNF–TNFRII axis may control the survival of MDSCs through upregulation of cellular FLICE-inhibitory protein (c-FLIP), leading to the inhibition of caspase-8 activity (32). Moreover, membrane-associated form of TNF (tmTNF) appears more potent than soluble TNF (sTNF) for MDSC activation (33). It was also reported that TNFRII is crucial for the suppressive activity of MDSCs, since myeloid cells without TNFRII failed to produce IL-6 and NO (34). Finally, in chronic inflammation experiments, it was found that TNF inhibits differentiation of myeloid cells and increases suppressive capacity of MDSCs. MDSCs from TNF-deficient mice failed to suppress T-cell proliferation, produced lower levels of iNOS, S100A8, S100A9, and RAGE (35).

Tumor microenvironment is orchestrated by a complex network of cells of both innate and adaptive immunity, which may contribute to the tumor progression, instead of inducing anti-tumor immune responses (36). MDSCs represent an important component of tumor microenvironment (23), which by activating different signaling pathways may induce survival and proliferation of tumor cells, suppress T-cell- and NK-cell-mediated anti-tumor immune responses, and promote angiogenesis and metastasis (9). Recent experimental data suggested that pro-inflammatory cytokines, such as TNF and IL-6, are necessary for the induction, expansion, and suppressive activity of MDSCs (33, 35, 37, 38). Therefore, we considered to address the impact of anti-cytokine therapy on tumor development and MDSC accumulation in a transplantable tumor model in mice. Pharmacological blockers of TNF and IL-6 are widely used in the clinic for treatment of various autoimmune disorders (39). Whether such long-term treatment may promote neoplasia in patients or, on the contrary, provide additional protection from emerging tumors is of high clinical relevance (40). In this study, we employed a unique experimental model to study the effects of TNF neutralization on tumor-induced expansion of MDSCs *in vivo*. Using pharmacological inhibition of human TNF in humanized mice (hTNF KI) with either etanercept or infliximab, we demonstrated that in mice transplanted with MCA 205 fibrosarcoma, MDSC accumulation and tumor growth were significantly diminished. Our study provides another example of pro-tumorigenic activity of endogenous TNF that involves MDSCs and may be useful for future validation of cell-type-specific anti-cytokine therapy, targeting TNF on myeloid cells (41).

## Materials and Methods

### Mice

Humanized TNF KI mice on C57Bl/6 background were recently described (41, 42). C57Bl/6 mice and hTNF KI mice were bred at Animal Breeding Facility of Shemyakin and Ovchinnikov Institute of Bioorganic Chemistry, Puschino, Moscow Region, Russia, housed under specific pathogen-free conditions on 12 h light/dark cycle at room temperature. For tumor injections, age- and sex-matched C57Bl/6 and hTNF KI mice were used at the age of 8–10 weeks. All manipulations with animals were carried out in accordance with recommendations in the Guide for the Care and Use of Laboratory Animals (NRC 2011), the European Convention for the protection of vertebrate animals used for experimental and other scientific purposes, Council of Europe (ETS 123), and “The Guidelines for Manipulations with Experimental Animals” (the decree of the Presidium of the Russian Academy of Sciences of April 02, 1980, no. 12000-496). All animal procedures were approved by Scientific Council of the Engelhardt Institute of Molecular Biology.

### Tumor Cell Lines

MCA 205 fibrosarcoma and EL4 T-cell lymphoma were cultured in RPMI 1640 medium supplemented with 10% FBS, l-glutamine (2 mM), 100 U/ml penicillin, and 100 μg/ml streptomycin in the T75 flasks. Following four to six passages, tumor cells were harvested, washed in PBS, and injected subcutaneously into the abdomen region of mice in the amount of 1 × 10^6^ cells in 200 μl. Tumor growth was monitored every 2–5 days, and tumor volume was calculated as (l × w × h) in cubic millimeter.

### TNF Blockers

Etanercept (Pfizer Ireland Pharmaceuticals, Ireland, J17413) and infliximab (Schering-Plough, Ireland, 3RMKA86001) were used to neutralize TNF. Mice were injected with 10 μg/g of etanercept or infliximab every 3 days. Control mice received PBS. Tumor cells were injected 1 week after the first injection of TNF inhibitors.

### Flow Cytometry Analysis

Single cell suspensions prepared from blood, spleen, and lymph nodes were stained with epitope-specific antibodies (eBioscience, Inc., San Diego, CA, USA): FITC-labeled Gr-1 (RB6-8C5), PE-labeled F4/80 (BM8), Pacific Blue-labeled B220 (RA3-6B2), Pe-Cy7-labeled Ly6C (HK 1.4), APC-labeled CD11b (M1/70), PerCP-Cy5.5-labeled CD45 (30-F11), and APC-Cy7-labeled viability dye. For blocking unspecific binding, anti-Fc gamma receptor antibodies (2.4G2) were used. For NO and ROS detection, carboxy-H_2_DCFDA (Thermo Fisher Scientific) and DAF-FM diacetate (Thermo Fisher Scientific) were used, respectively, each at a final concentration of 1 μM. Data were acquired with FACSCanto II Cytometer (BD Biosciences) and analyzed with FlowJo software. Gating strategies are summarized in the supplementary figures for MDSCs in the blood (Figure S1A in Supplementary Material), MDSCs in the spleen and the lymph nodes (Figure S1B in Supplementary Material), T-cell proliferation assay (Figure S2A in Supplementary Material), and NO and ROS in the blood (Figure S2C in Supplementary Material).

### Real-time Quantitative RT-PCR Analysis

Total RNA was isolated using the TRIzol Reagent (Invitrogen, Carlsbad, CA, USA), according to the manufacturer’s protocol. Reverse transcription was performed using 1 μg total RNA and oligo(dT)_18_ primers with RevertAid first strand cDNA synthesis kit (Thermo Scientific, USA), according to the manufacturer’s protocol. Real-time quantitative PCR was performed using qPCRmix-HS SYBR kit (Evrogen, Moscow, Russia) on the Applied Biosystems 7500 Real-Time PCR System (Applied Biosystems, Foster City, CA, USA). The following primers were used: *Actb*, F: 5′-GACCTCTATGCCAACACAGT, R: 5′-AGAAAGGGTGTAAAACGCAG; *S100A9*, F: 5′-TTAGCCTTGAAGAGCAAG AAGATGG, R: 5′-AGCTCAGCTGATTGTCCTGGT; *Adam17*, F: 5′-GGCCGG AAACGAGTTAAGCC, R: 5′-AGCTTCTCAAGTCGCGGATG; *Nos2*, F: 5′-GTCA ACTGCAAGAGAACGGAGA, R: 5′-TCTGTGCTGTCCCAGTGAGG; *Bcl2l1*, F: 5′-TGGAGTAAACTGGGGTCGCA, R: 5′-TCCACAAAAGTGTCCCAGCC; *Arg1*, F: 5′-CTCTGGGAATCTGCATGGGC, R: 5′-GGCCTTTTCTTCCTTCCCAGC; *Tgfb1*, F: 5′-TGCTGACCCCCACTGATACG, R: 5′-GTTTGGGGCTGATCCCGTTG; *S100a8*, F: 5′-CTTCAAGACATCGTTTGAAAGG, R: 5′-ATTCTTGTAGAGGGCATGGT; *Il10*, F: 5′-GACAATAACTGCACCCACTTCC, R: 5′-AACCCAAGTAACCCTTAAAGTCC; and *Il6*, F: 5′-GTGGAAATGAGAAAAGAGTTGTGC, R: 5′-GGAGAGCATTGGAAATTGGGGT. Amplifications were performed using the following program: preheating stage at 95°C for 10 min, 40 cycles at 95°C for 15 s, annealing at 61°C for 30 s, and extension at 72°C for 20 s. Relative expression of target genes was determined according to ΔΔCt with normalization to *Actb* expression.

### T-Cell Proliferation Assay

T-cells were isolated from spleens of naive mice using CD4 (L3T4) MicroBeads, according to the manufacturer’s protocol (Miltenyi Biotec, Germany). T-cells were labeled with 5 mM CFSE (Molecular Probes, USA) for 15 min at 37°C, washed three times with cold RPMI, and diluted in 96-well round-bottom plates at concentration 4 × 10^5^ cells in RPMI 1640 medium supplemented with 10% FBS, l-glutamine (2 mM), 100 U/ml penicillin, 100 μg/ml streptomycin, 10 mM Hepes, 50 μM b-ME, MEM (Thermo Fisher Scientific, 11130-051), and sodium pyruvate (1 mM) in each well. These cells were cocultured with 2 × 10^6^ purified splenic MDSCs from tumor-bearing mice undergoing etanercept, infliximab, or PBS treatment. CD11b^+^Gr-1^+^ cells were purified from the spleens of tumor-bearing mice using MDSC isolation kit, according to the manufacturer’s protocol (Miltenyi Biotec, Germany). For stimulation of T-cell proliferation, we used anti-CD3 (clone 145-2C11) and anti-CD28 (clone 37.51) antibodies in final concentrations of 1 μg/ml and 6 ng/ml, respectively. After 72 h, cells were collected and analyzed by flow cytometry (Figure S2A in Supplementary Material).

### Statistical Analysis

Statistical analysis was performed using GraphPad Prism software (version 6, San Diego, CA, USA). Two-tailed unpaired Student’s *t*-test was used for comparison of two independent data samples and determination of the degree of reliability. The data were obtained in at least three independent experiments and presented as the mean ± SD. *P* values <0.05 were considered to indicate statistical significance.

## Results

### Systemic TNF Ablation with Etanercept Efficiently Reduces MCA 205 Tumor Growth and MDSC Accumulation in C57Bl/6 Mice

**Figure 1:**
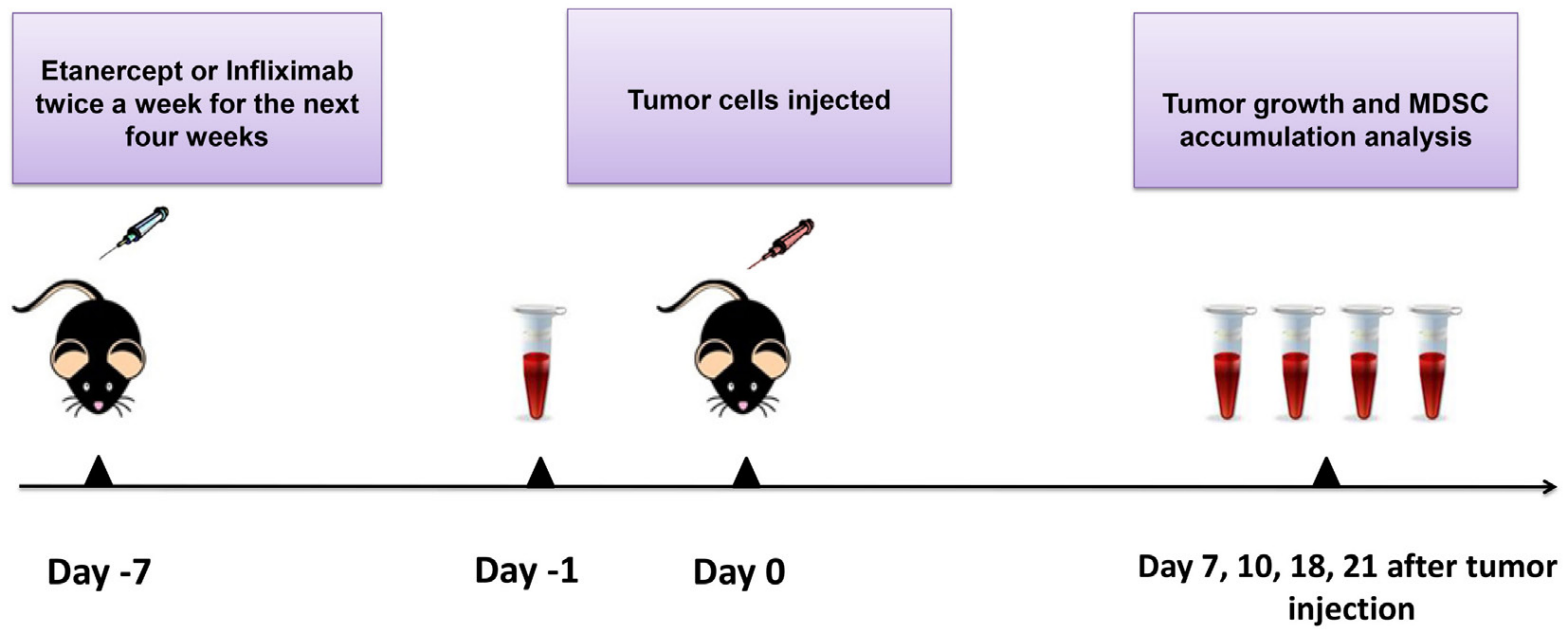
**Scheme of the experiment**. Mice were injected i.p. with 10 μg/g of etanercept, infliximab, or PBS in the volume of 200 μl twice a week during 4 weeks of the experiment. Exponentially growing 1 × 10^6^ MCA 205 tumor cells were injected 1 week after the initiation of anti-TNF therapy. Tumor growth was monitored every 2–3 days, and tumor volume was calculated as (l × w × h). Peripheral blood was drawn from the recipient mice prior to tumor cells inoculation (day −1) and then every 4–7 days. Blood samples were analyzed by flow cytometry for MDSC accumulation. After 21 days of tumor growth, mice were euthanized and spleens, lymph nodes, and tumors were collected for flow cytometry analysis and MDSC separation.

**Figure 2:**
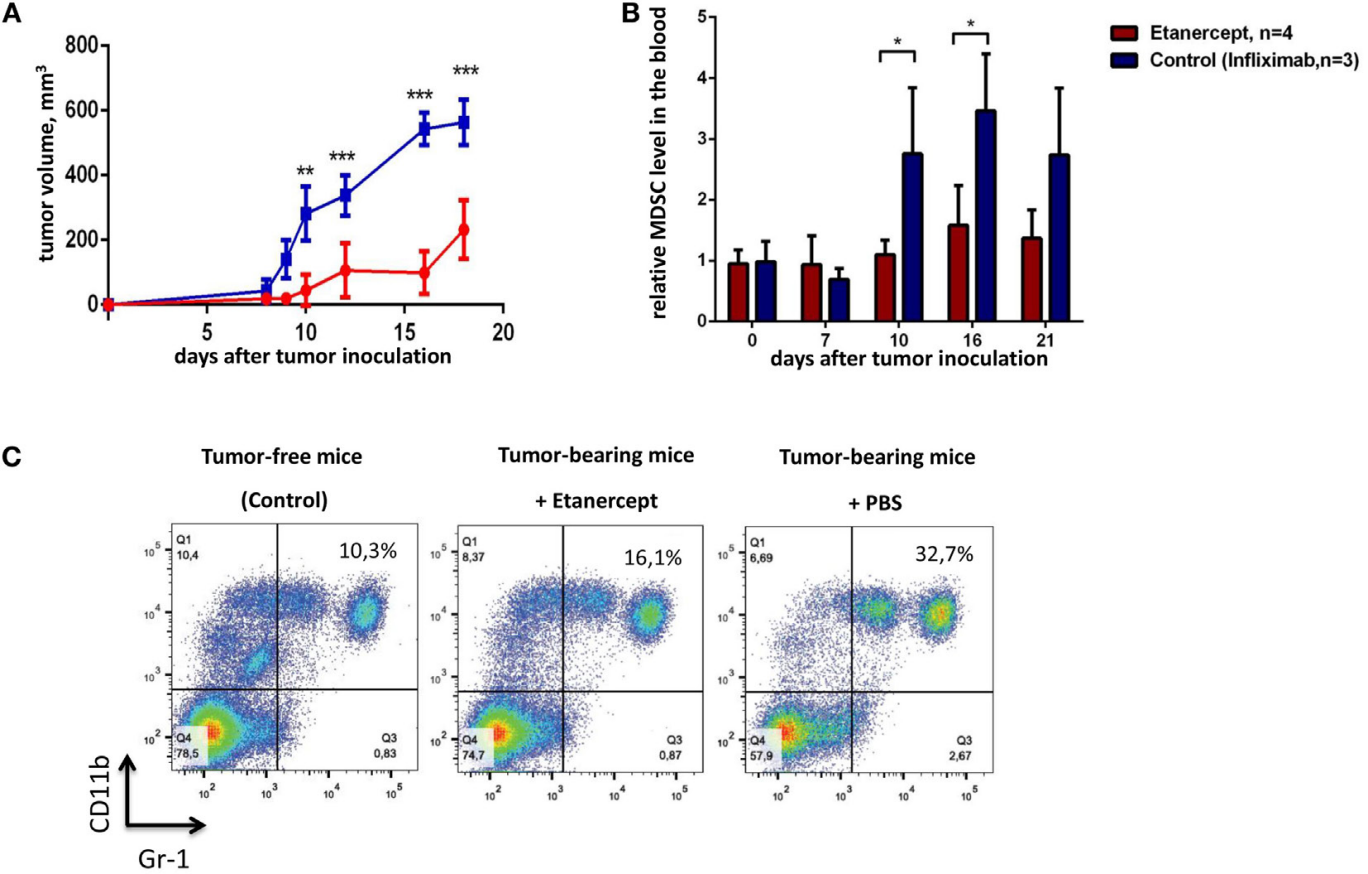
**Etanercept efficiently reduces transplantable tumor growth and MDSC accumulation in C57Bl/6 mice**. **(A)** Tumor growth in control mice (blue) and mice undergoing treatment with etanercept (red). Each line represents the growth curve of mean tumor volume ±SD. **(B)** MDSC accumulation in the blood. Each bar represents mean relative MDSC level in the blood of tumor-bearing mice normalized to tumor-free mice ± SD. **(C)** Representative dot plots for MDSC staining in the blood from mice without tumors (left), etanercept (middle), or PBS-treated (right) tumor-bearing mice on day 16 after tumor cells inoculation. Cells were first gated as VD^−^CD45^+^, and then CD11b^+^Gr-1^+^ cells were defined as MDSCs (with Gr-1 antibody clone RB6-8C5 recognizing both Ly6G and Ly6C epitopes). Data are representative of two independent experiments with three to five mice per each group. *p < 0.5, **p < 0.01, and ***p < 0.001.

TNF is important for MDSC development, in turn, MDSCs play a crucial role in tumor progression (9, 35). Genetic studies suggested that TNF may play a pro-tumorigenic role in skin carcinogenesis model (27). Thus, we hypothesized that prolonged *in vivo* blockade of TNF with pharmacological agents may result in anti-tumor effects. To test this hypothesis, we evaluated the ability of clinically used TNF blockers to prevent transplantable tumor growth in mice. Our initial experiments were carried out in C57Bl/6 mice using etanercept, a soluble fusion protein of human p75 TNF receptor and Fc portion of IgG1 antibody, as an inhibitor of murine TNF, because it is the only clinically available blocker that binds to murine TNF (43). We have chosen MCA fibrosarcoma cell line, since a potent anti-tumor effect of TNF *in vivo* was originally discovered on methyl-cholantrene-induced tumors (25). Specifically, we used transplantable tumor cell line, MCA205 fibrosarcoma, injected into C57Bl/6 mice, because the resulting tumors are known to be dependent on MDSC accumulation (32). As a control reagent in this first set of experiments, we used infliximab, a chimeric monoclonal antibody against human TNF, which does not bind murine TNF but has the same Fcγ-domain as etanercept (44). The scheme of the experiment is shown in Figure 1. We first examined the growth kinetics of MCA 205 fibrosarcoma (Figure 2A) in C57Bl/6 recipients. Mice treated with infliximab or PBS rapidly developed tumors with the similar kinetics (only infliximab group is shown as a control on Figure 2). Importantly, mice injected with etanercept demonstrated a significant reduction in tumor volume starting from day 10 after tumor inoculation (Figure 2A). We then evaluated the effects of systemic TNF ablation on MDSC accumulation by treating mice with etanercept (Figures 2B,C) and compared MDSC levels in the blood of tumor-bearing and tumor-free mice. As expected, mice under etanercept therapy accumulated significantly less MDSCs in the blood compared to mice under infliximab or PBS treatment, whereas tumor-free mice had very low MDSC levels (Figure 2B and data not shown). Moreover, we observed differences in MDSC levels starting from day 10, when the differences in tumor growth were also significant. Taken together, these data show that TNF neutralization might reduce tumor growth and MDSC accumulation in a transplantable MCA tumor model in mice.

### Treatment with Infliximab or Etanercept Efficiently Reduces Tumor Growth and MDSC Accumulation in Humanized TNF KI Mice

**Figure 3:**
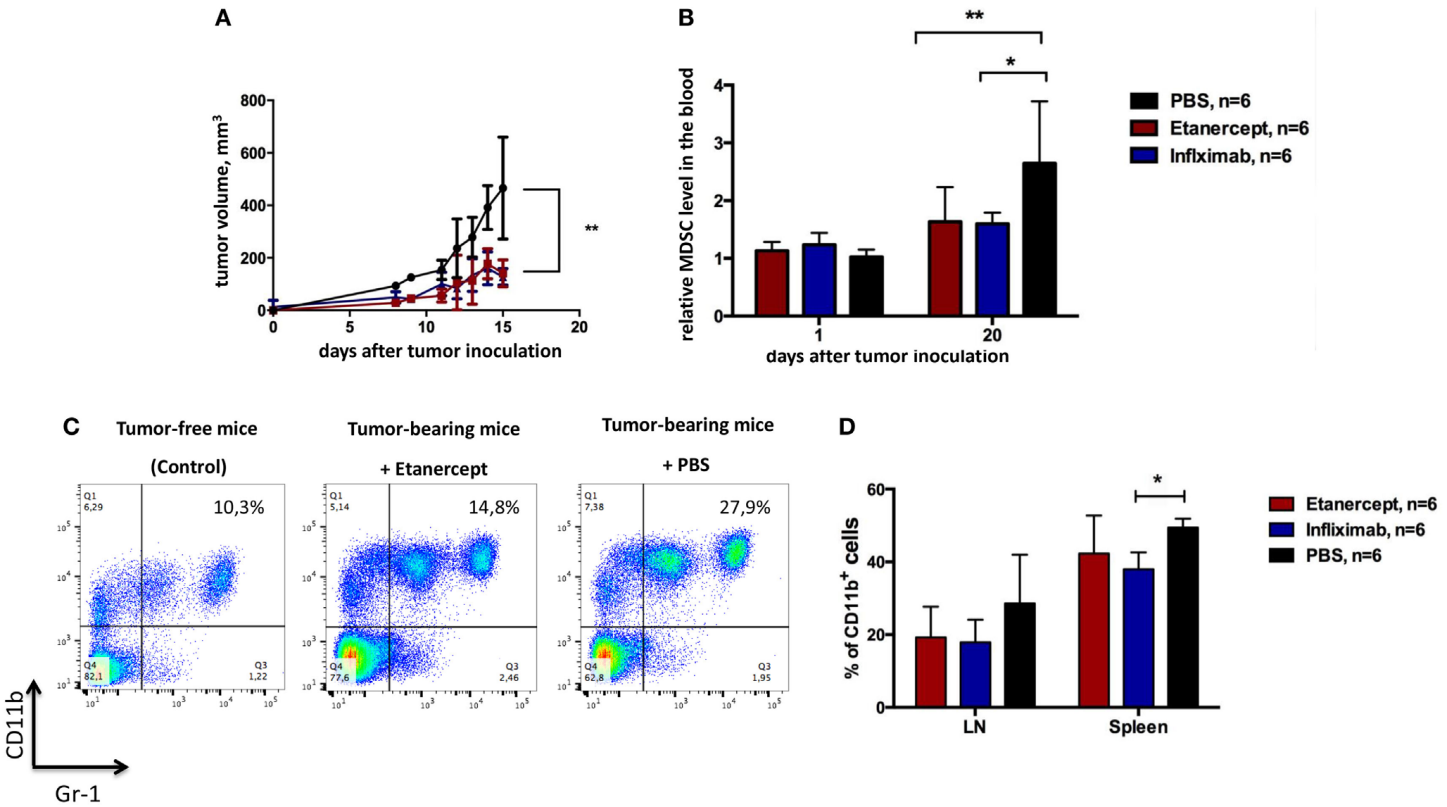
**Infliximab or etanercept efficiently reduce tumor growth and MDSC accumulation in hTNF KI mice**. **(A)** Tumor growth in hTNF KI mice undergoing treatment with PBS (black), etanercept (red), or infliximab (blue). Each line represents the growth curve of mean tumor volume ±SD. **(B)** MDSC accumulation in the blood of tumor-bearing hTNF KI mice undergoing treatment with PBS (black), etanercept (red), or infliximab (blue). Each bar represents mean relative MDSC level in the blood of tumor-bearing mice normalized to tumor-free mice ±SD. **(C)** Representative dot plots for MDSC staining in the blood from mice without tumors (left), anti-TNF (middle), or PBS-treated (right) mice with tumors on day 16 after tumor cells inoculation. Cells were first gated on VD−CD45^+^. MDSCs were defined as CD11b^+^Gr-1^+^ cells (with Gr-1 antibody clone RB6-8C5 recognizing both Ly6G and Ly6C epitopes). **(D)** MDSC accumulation in the spleens and peripheral lymph nodes of tumor-bearing mice three weeks after tumor inoculation. Graph shows relative numbers of Ly6C^+^Ly6G^+^cells among CD11b^+^ cells. Cells were gated as VD-CD45^+^B220^–^. Data are representative of more than three independent experiments with five to six mice per each group; *p < 0.5 and **p < 0.01.

One important difference between etanercept and antibody-based anti-TNF drugs (such as infliximab, adalimumab, and others) is that the former may also bind and neutralize soluble LTα_3_ (45). Because several non-redundant functions of sLTα_3_ were reported, for instance, the role of LTα_3_ produced by innate lymphoid cells in the gut (46), we wanted to make sure that the effects of etanercept in our tumor model (including results presented in Figure 2) were due to neutralization of TNF and not to soluble LTα_3_. To this end, we compared the effects of anti-TNF therapy on tumor growth using humanized knock-in mice, which produce human TNF instead of murine TNF (further referred to as hTNF KI mice) (41, 42). This model allowed us to compare therapeutical effects of various human TNF blockers *in vivo*, in our case – etanercept and infliximab. First of all, tumor growth (Figures 2A and 3A) and the accumulation of MDSCs (Figures 2B and 3B) in tumor-bearing hTNF KI mice were comparable to those in wild-type mice, in both cases MDSCs reached 30–40% in the blood 3 weeks after MCA 205 tumor cells inoculation. We then compared the effects of TNF blockade with either etanercept or infliximab on MCA 205 growth and found that both drugs efficiently and comparably inhibited tumor growth (Figure 3A), indicating that anti-tumor effects were most likely due to TNF and not to sLTa neutralization. Three weeks after inoculation, the tumor volume in mice undergoing anti-TNF treatment reached only 200 mm^3^, as compared to 500–600 mm^3^ in the control group. Histological analysis revealed infiltration of the tumor tissue by myeloid cells in all three experimental groups (Figure S3 in Supplementary Material). We also observed a significant reduction in MDSC levels in the blood of mice treated with either of the two blockers (Figure 3B). MDSC accumulation in control mice reached 30–40% of total leukocytes in the blood, while in mice treated with etanercept or infliximab it only reached 15–20% (Figure 3C). Tumor-free mice in each group, despite receiving TNF blockers, showed the same frequency of CD11b^+^Gr-1^+^ myeloid cells, at approximately 8–10% of total blood leukocytes (Figure 3C). We also followed accumulation of MDSCs in the periphery: in lymph nodes and in the spleen, and found that the frequency of MDSCs was higher in PBS-treated group (Figure 3D). These data indicated that TNF plays a crucial role in MCA205 tumor growth and MDSC accumulation.

### Systemic TNF Inhibition Affects Suppressive Functions of MDSCs

**Figure 4:**
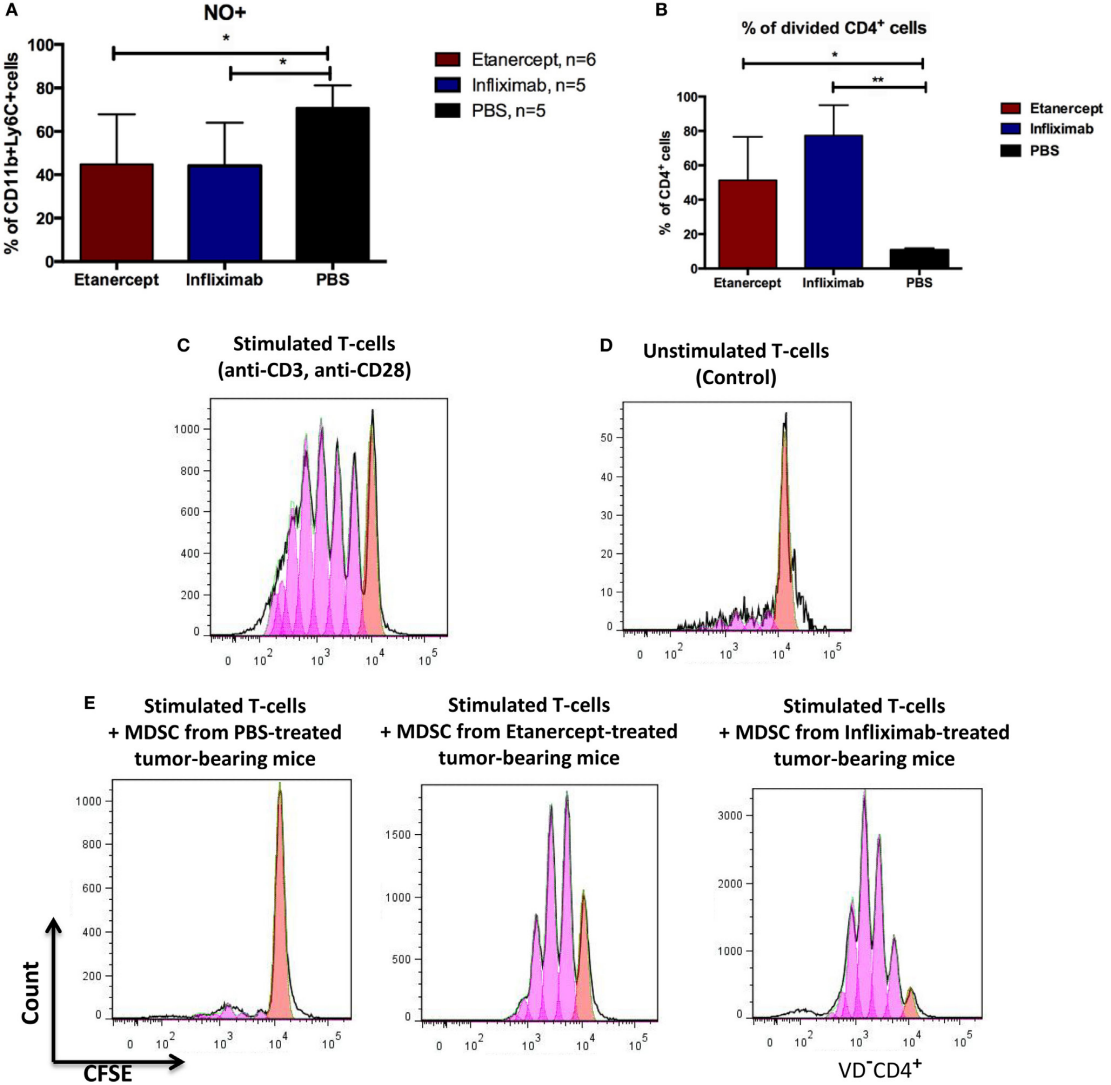
**Infliximab or etanercept efficiently inhibit suppressive functions of MDSCs**. **(A)** Blood MDSCs of tumor-bearing hTNF KI mice undergoing treatment with etanercept (red) or infliximab (blue) have reduced levels of NO compared to PBS-treated mice (black). Data are representative of two independent experiments. **(B)** Purified splenic MDSCs from tumor-bearing hTNF KI mice undergoing systemic TNF ablation with etanercept (red) or infliximab (blue) fail to suppress T-cell proliferation. MDSCs and T-cells were co-cultured at ratio 5:1, 2 × 10^6^ and 4 × 10^5^ cells, respectively. **(C)** *In vitro* proliferation of purified CFSE-labeled T-cells from the spleens of naive mice after three days of stimulation with anti-CD3 and anti-CD28 antibodies or **(D)** unstimulated T-cells. **(E)** CFSE-labeled T-cells from naive mice after three days of stimulation with anti-CD3 and anti-CD28 antibodies in the presence of purified splenic MDSCs from tumor-bearing mice treated with PBS (left), etanercept (middle), or infliximab (right). Pink and red peaks were defined as non-proliferating and proliferating cells, respectively. Cells were gated as VD^−^CD4^+^. Data are representative of three independent experiments with each group represented by six mice, and MDSCs were isolated from pooled splenocytes from two to three mice. *p < 0.5 and **p < 0.01.

To study the impact of systemic TNF ablation on the functional properties of MDSCs, we examined ROS and NO production *in vivo* by blood MDSCs of tumor-bearing hTNF KI mice undergoing treatment with TNF inhibitors. We detected a significant reduction in NO in CD11b^+^Ly6C^+^ myeloid cells in the blood of both etanercept- and infliximab-treated mice as compared to PBS-treated hTNF KI tumor-bearing mice (Figure 4A), whereas significant difference in ROS production was only detected in etanercept-treated tumor-bearing hTNF KI mice (Figure S2B in Supplementary Material). Furthermore, to address possible impact of anti-TNF treatment on MDSC functions, we evaluated suppressive activity of myeloid cells on T-cell proliferation using a co-culture of CFSE-labeled T-cells and purified MDSCs in the presence of agonistic anti-CD3 and anti-CD28 antibodies. MDSCs were isolated from the spleens of hTNF KI tumor-bearing mice undergoing treatment with anti-TNF blockers or with PBS, as control. After 3 days of co-culture, we determined the label distribution in the population of CD4^+^ T-cells (Figure 4B). As expected, purified MDSCs isolated from PBS-treated tumor-bearing mice completely suppressed T-cell proliferation (Figures 4B,E, left). Strikingly, MDSCs isolated from tumor-bearing mice under infliximab or etanercept treatment were not able to prevent T-cell proliferation indicating that their suppressive function was compromised (Figures 4B,E, middle and right). Unstimulated and stimulated T-cells in the absence of MDSCs are shown as control stainings in Figures 4C,D). We also analyzed gene expression and evaluated their suppressive activity *ex vivo*. For this, we isolated MDSCs from the spleens of wild-type tumor-bearing mice after etanercept administration using magnetic bead separation (Figure S4A in Supplementary Material). We then performed gene expression analysis to see how anti-TNF treatment may affect the transcriptome of MDSCs (Figure S4B in Supplementary Material). We specifically looked for genes that encoded factors known to play an important role in MDSC function and found that at least some of them were indeed affected by systemic anti-TNF treatment. In particular, we observed significant reduction in the expression of *il10*, *adam17*, and *tgfb*, while the expression of *arginase-1* gene was increased (Figure S4B in Supplementary Material). Taken together, our data demonstrated that systemic TNF inhibitors may not only reduce MDSC numbers but also affect their suppressive function.

## Discussion

Tumor microenvironment plays an important role in tumor growth and in resistance to therapies, in particular, through suppression of anti-tumor immune responses and by promoting angiogenesis, metastasis, and survival of cancer cells (47). Although certain mechanisms governing cross talk between microenvironment and tumor cells have been previously elucidated, the role of pro-inflammatory cytokines, such as TNF, IL-6, and IL-1, is not completely understood (48, 49). MDSCs represent a component of tumor microenvironment, which provides suppressive effects on immune cells, such as cytotoxic T-cells and NK-cells (23). Recently, it was shown that TNF is important for MDSC survival, expansion, and function (32, 33, 35). Role of TNF is particularly interesting because TNF, as its name suggests, has a necrotizing anti-tumor activity in MCA sarcoma model in mice (25) that was reproduced with recombinant human TNF (50) and also because these anti-sarcoma effects are used clinically in the isolated limb perfusion setting (51). However, in other experimental models in mice and also in patients, TNF was reported to play a pro-tumorigenic role, and thus its therapeutic blockade may prove beneficial (40, 52). In our study, we demonstrated that systemic pharmacologic TNF ablation leads to the delay in transplantable tumor growth of MCA 205 fibrosarcoma, accompanied by decreased accumulation of MDSCs.

**Figure 5:**
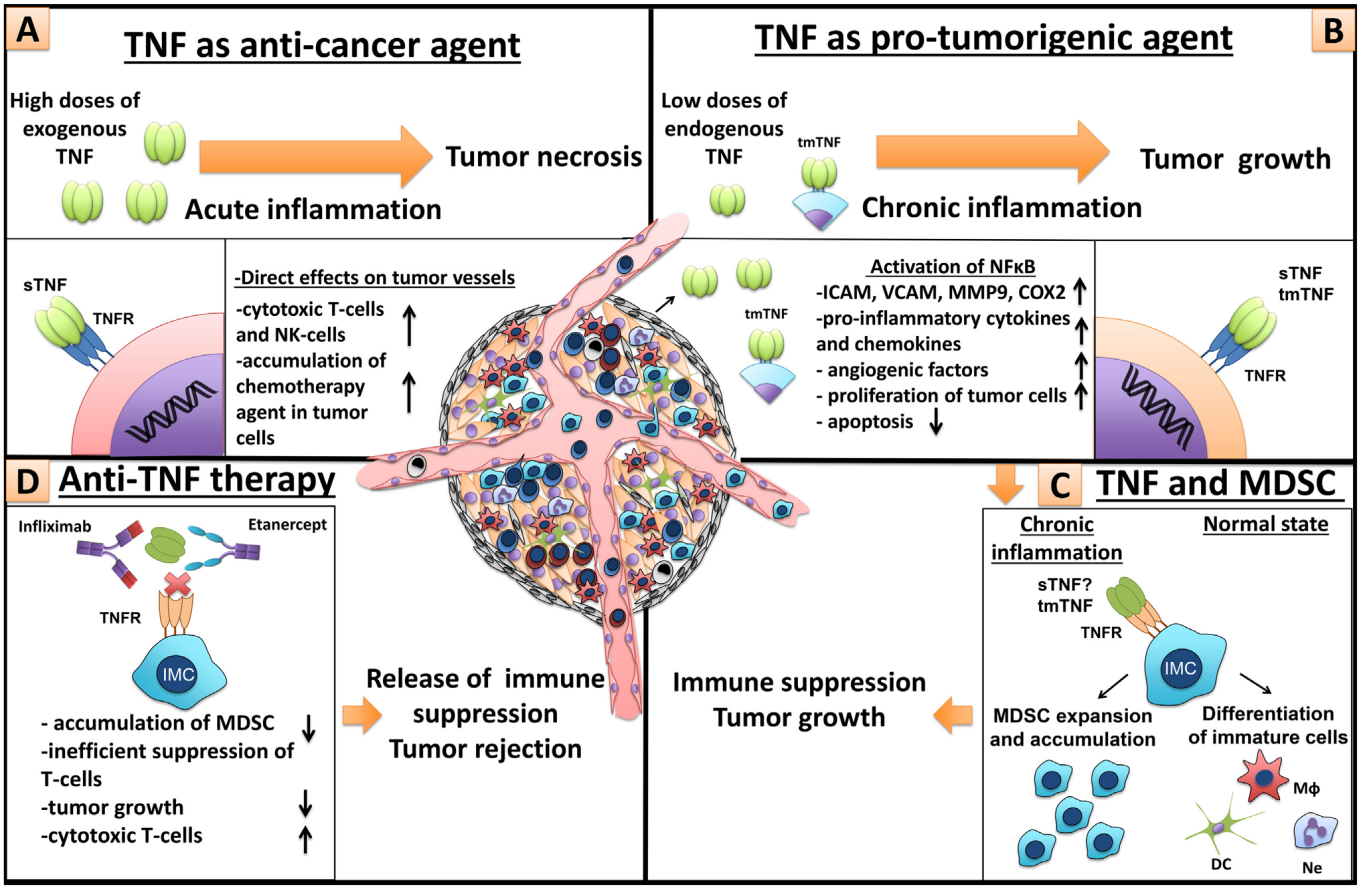
**Dual role of TNF in cancer and MDSCs**. **(A)** TNF as anti-cancer agent. High doses of exogenous TNF induce tumor necrosis (25). However, necrotizing effect of TNF is not due to cytotoxic killing of tumor cells but because of direct action of TNF on endothelial cells, which leads to significant destruction of tumor-associated vessels and tumor rejection (56). Furthermore, TNF is an important molecule for anti-tumor immunity by cytotoxic NK- and T-cells (57, 58). In patients, TNF alone is inefficient, but in combination with chemotherapy, it increases tissue concentration of chemotherapy drugs, through the increase of blood vessel permeability, and is used in isolated perfusion procedure for the sarcoma treatment of the limb (59). **(B)** TNF as a pro-tumorigenic molecule. Binding of TNF with TNFR on endothelial cells and cancer-associated fibroblasts leads to NFκB activation and upregulation of chemokines, adhesion molecules, growth factors, and pro-inflammatory cytokines, resulting in increased angiogenesis, inflammation, and recruitment of immune cells (60, 61). TNF through the same signals activates proliferation of tumor cells and induces the release of different factors by tumor cells, such as metalloproteinases and VEGF, resulting in angiogenesis and tumor niche remodeling (62). **(C)** TNF and MDSCs. TNF and other pro-inflammatory cytokines, produced by tumor cells and tumor microenvironment, sustain chronic inflammation (63). During chronic inflammation myeloid cells do not differentiate into mature macrophages, neutrophils, and dendritic cells but rather accumulate as immature cells with suppressive capacity, so-called MDSCs (1). TNF is crucial for MDSCs, due to its direct effects on myeloid cells (33–35). MDSCs suppress anti-tumor immunity driving tumor development (9). **(D)** Anti-TNF therapy. Neutralization of TNF by etanercept or infliximab may result in tumor delay, decrease of MDSC accumulation, inefficient T-cell suppression, and increase of cytotoxic T-cells.

The reasons for these contrasting effects of endogenous versus systemically administered TNF are not fully understood. It is known that hemorrhagic necrosis of tumors is not due to direct TNF cytotoxicity on tumor cells but rather due to acute effects on tumor vasculature that is a part of tumor microenvironment (53) (Figure 5A). It is generally believed that such acute activating effects of TNF are very fast and are mediated by TNFRI. On the other hand, local effects of endogenous TNF, released from tumor cells and from tumor microenvironment, may be local and long-lasting and they could be mediated by both TNF receptors, with TNF–TNFRII axis having a distinct role because it requires tmTNF and cell-to-cell contacts (54). The downstream effects of such signaling may lead to the expression of pro-inflammatory cytokines, chemokines, and adhesion molecules resulting in chronic inflammation – one of the hallmarks of cancer development (55) (Figure 5B). In addition to this, TNF promotes expansion of MDSCs, which have pro-tumorigenic role and also accumulate during chronic inflammation (32, 33, 35) (Figure 5C). As already discussed, TNF therapy has found only limited clinical application in cancer treatment, while anti-TNF therapy is widely used in the treatment of autoimmune diseases. Could such systemic and often long-term TNF blockade predispose autoimmune patients to cancer? Or on the contrary, could anti-cytokine therapy provide a protection against emerging tumors? These are types of questions that we want to address in animal models.

In order to compare possible *in vivo* anti-tumor effects of TNF blockers used in clinic, we utilized an experimental model of humanized (hTNF KI) mice, endogenously producing human but not mouse TNF (41, 42). We found that after injection of tumor cells, hTNF KI mice develop MCA 205-derived tumors comparably with C57Bl/6 mice. TNF neutralization by either of the two blockers, infliximab or etanercept, resulted in comparable reduction of tumor volume in hTNF KI mice and also correlated with reduced frequency of MDSCs, which may play a pro-tumorigenic role (Figure 5D). This clearly suggests that the effects of etanercept administration are due to TNF and unlikely due to soluble LTα_3_ blockade. This is an important observation, since the role of lymphotoxin in carcinogenesis has been previously described (64–68). Furthermore, our experiments suggest that TNF inhibition affects MDSCs by altering their gene expression. Specifically, purified CD11b^+^Gr-1^+^ cells, isolated from C57Bl/6 mice undergoing etanercept treatment, showed decreased expression levels of genes that encode anti-inflammatory cytokine, such as TGF-β and IL-10, which are necessary for polarization of M2 macrophages (69), differentiation of Tregs, capable of functional inhibition of cytotoxic cells (20–22). Specifically, we detected a reduced expression of the gene encoding ADAM17, which may cleave off CD62L from the surface of T-cells, providing MDSCs with the ability to prevent activation of naive T-cells by blocking their migration to the sites of inflammation (70). The same protease converts membrane-bound forms of pro-inflammatory cytokines and their receptors, such as IL-6R and TNF, into secreted soluble forms (71). Interestingly, in MDSCs from mice receiving anti-TNF treatment, we detected an increase in the expression level of *arginase-1* gene. This enzyme facilitates suppressive activity of MDSCs through degradation of arginine (14). Thus, these changes in gene expression could affect the function of myeloid cells and may be important for anti-tumor immunity. To address the question of how TNF blockade could affect suppressive capacity of MDSCs, we looked at ROS and NO production by blood MDSCs *in vivo* and also tested the ability of MDSCs to inhibit proliferation of T-cells in a functional assay *ex vivo*. We found that purified splenic MDSCs from hTNF KI tumor-bearing mice, undergoing infliximab or etanercept treatment, failed to suppress T-cell proliferation, whereas MDSCs from the control PBS-treated tumor-bearing mice had the expected suppressive activity. This supports our *in vivo* data concerning significantly reduced level of NO in blood MDSCs of mice receiving either etanercept or infliximab. These findings imply that TNF inhibition not only reduces accumulation of MDSCs but also affects their suppressive capacity. Overall, our data support the notion that MDSCs may be a target for anti-tumor therapy in patients and it also highlights TNF as a mediator with non-redundant pro-tumorigenic functions. One possible avenue in anti-cytokine therapy being developed in our laboratory is to neutralize pro-inflammatory cytokines in cell-restricted fashion (41). For using such a strategy in cancer treatment, cellular sources of pro-tumorigenic cytokine in each particular case should be first identified.

## Author Contributions

MD, K-SA, and SN designed the research; ZQ contributed to the new experimental approach; K-SA, VG, MN, RZ, and MD performed the experiments; K-SA and MN analyzed the data; K-SA, MN, MD, ZQ, and SN discussed the data; and K-SA, MD, and SN wrote the paper.

## Conflict of Interest Statement

The authors declare that the research was conducted in the absence of any commercial or financial relationships that could be construed as a potential conflict of interest.
