# Supplementary material for: TNF Neutralization Results in the Delay of Transplantable Tumor Growth and Reduced MDSC Accumulation
Source: Front Immunol. 2016 Apr 19;7:147. doi: 10.3389/fimmu.2016.00147 (PMC4835443; doi:10.3389/fimmu.2016.00147)
Supplement: Supplementary file 1 [file Presentation_1.PDF]

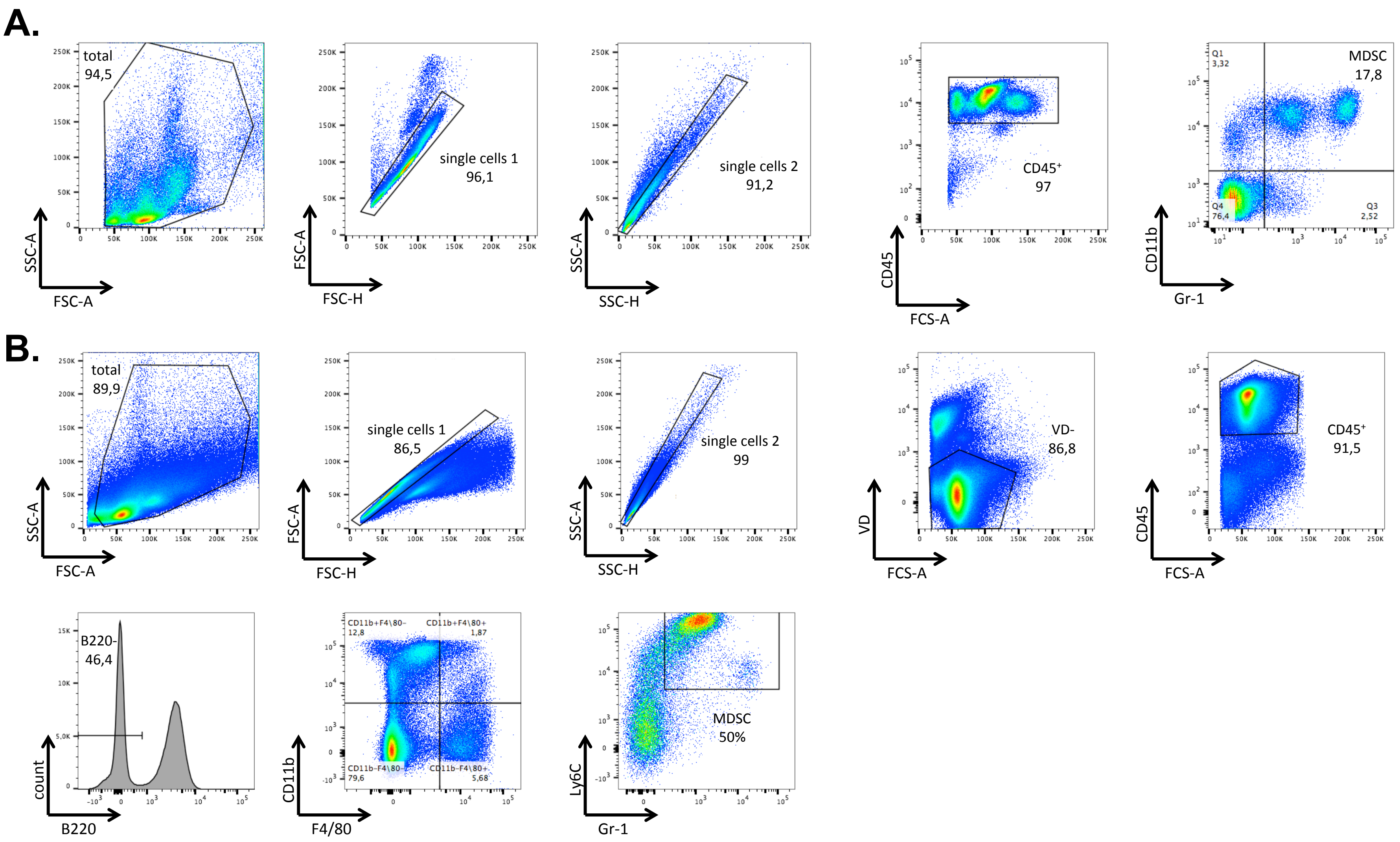

Supplementary figure 1. Gating strategy for flow cytometry analysis of MDSC in the blood and in the spleen or lypmh nodes. Single cell suspensions prepared from blood (A), spleen and lymph nodes (B) were stained with epitope-specific antibodies (eBioscience, San Diego, CA, USA): FITC-labeled Gr-1, PE-labeled F4/80, Pacific Blue-labeled B220, Pe-Cy7-labeled Ly6C, APC-labeled CD11b, PerCP-Cy5.5-labeled CD45, APC-Cy7-labelled Viability Dye. For blocking unspecific binding anti-Fc gamma receptor antibodies (2.4G2) were used. Data was acquired with Facs Canto II Cytometer (BD Biosciences) and analyzed with FlowJo software.

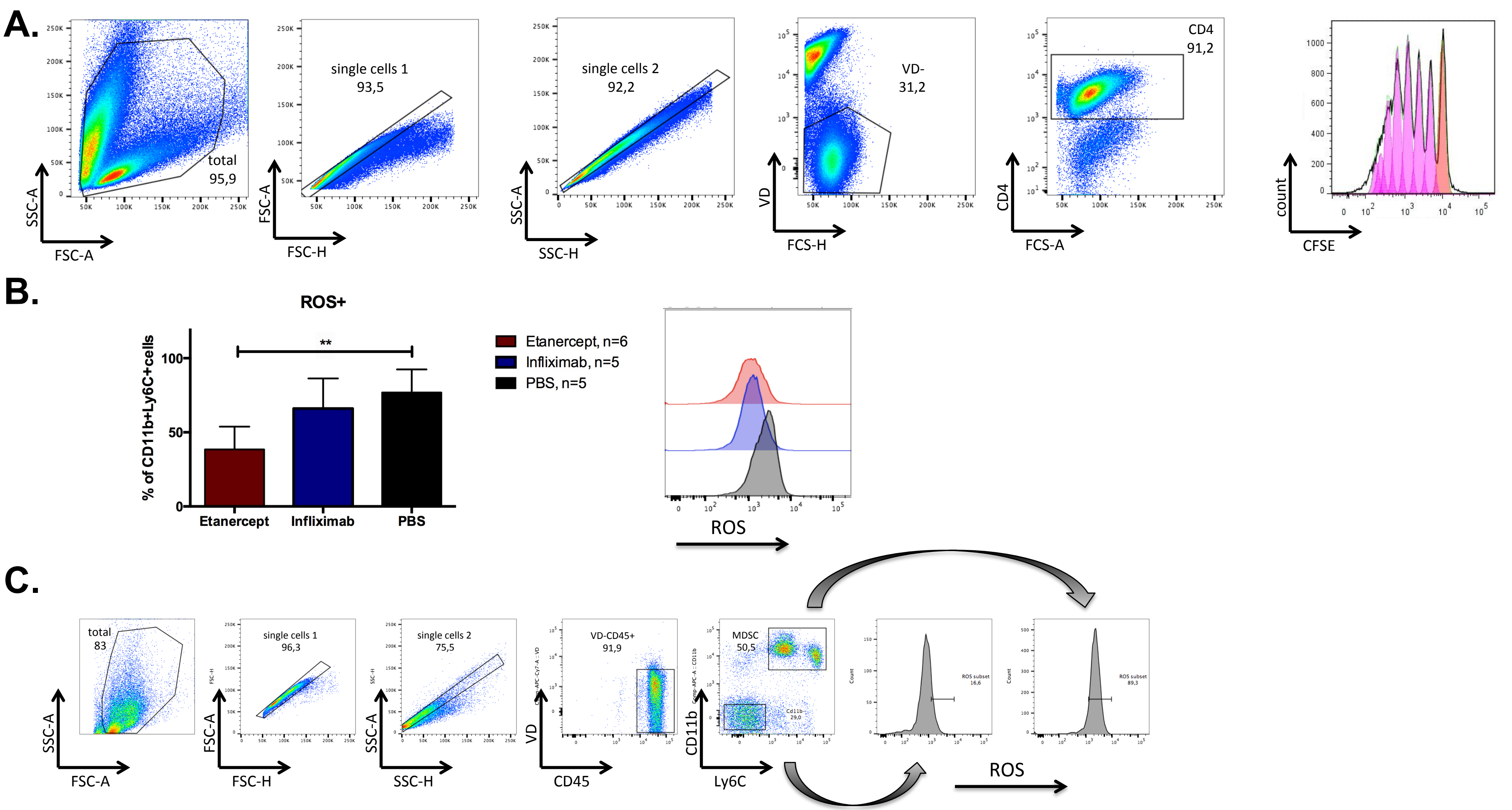

Supplementary figure 2. Gating strategy for flow cytometry analysis of CFSE-labeled CD4$^+$ cells and ROS, NO levels in the blood of mice 2-3 weeks after tumor inoculation . A) T-cells were isolated from the spleen of naïve mice with CD4-(L3T4)-MicroBeads according to manufacturer's protocol (Miltenyi Biotec, Germany). T-cells were labeled with 5mM CFSE (Molecular Probes, USA) for 15 minutes at 37^{0}C, washed 3 times with cold RPMI and diluted in 96-well round-bottom plates at concentration 3x10^5 cells in RPMI 1640 medium supplemented with 10% FBS, L-Glutamine (2 mM), 100 U/ml penicillin, 100 mcg/ml streptomycin, 10 mM Hepes, 50 mcM b-ME, MEM (Thermo Fisher Scientific, 11130-051), Sodium Pyruvate (1mM) in each well. These cells were co-cultured with 2.5x10^6 purified splenic MDSC from tumor-bearing mice undergoing Etanercept, Infliximab or PBS treatment. For stimulation of T-cell proliferation we used anti-CD3 (clone 145-2C11) and anti-CD28 (clone 37.51) antibodies in final concentration 1 mcg/ml and 6 ng/ml respectively. After 72 hours cells were collected and analyzed by flow cytometry. B) ROS production by CD11b+Ly6C+ cells in the blood of tumor-bearing hTNF KI mice undergoing treatment with PBS (black), Etanercept (red) or Infliximab (blue). Each bar represents mean level of Carboxy-H_2DCFDA $^+$CD11b $^+$Ly6C$^+$ in the blood of tumor-bearing mice 2-3 weeks after tumor inoculation. C) Single cell suspensions prepared from blood were stained with epitope-specific antibodies (eBioscience, San Diego, CA, USA):, APC-Cy7-labelled Viability Dye, PerCP-Cy5.5-labelled CD45, Pe-Cy7-labeled Ly6C, APC-labeled CD11b. For blocking unspecific binding anti-Fc gamma receptor antibodies (2.4G2) were used. Levels of ROS and NO were measured by flow cytometry using Carboxy-H_2DCFDA (ThermoFisher Scientific) and DAF-FM Diacetate (ThermoFisher Scientific) at a final concentration 1 µM respectievly. Data was acquired with FACS Canto II Cytometer (BD Biosciences) and analyzed with FlowJo software.

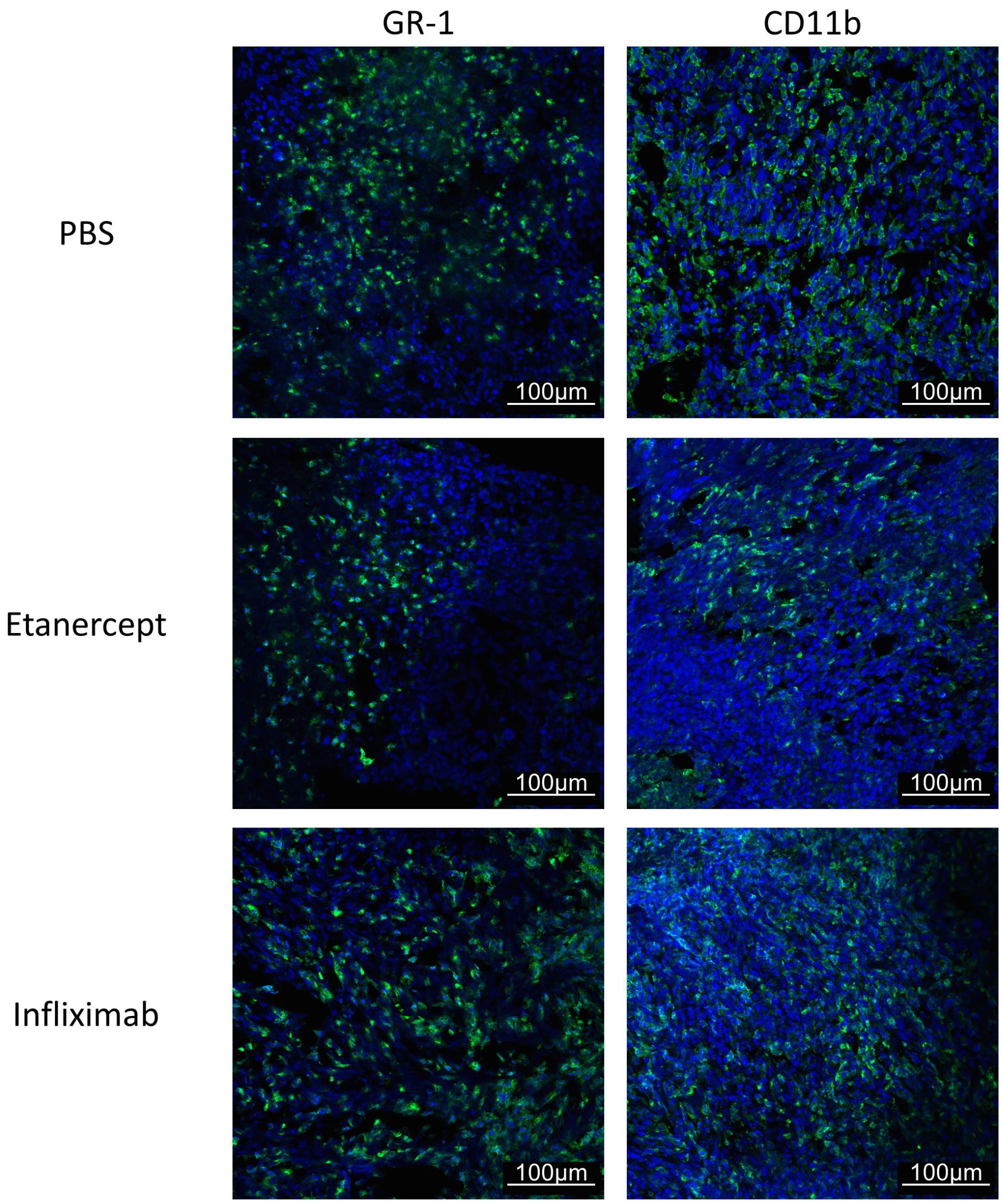

Supplementary figure 3. Immunohistochemical analysis of myeloid cells at the tumor site. Histological staining of tumors from PBS- (top), Etanercept- (middle) or Infliximab- (bottom) treated tumor-bearing mice for Gr-1 (left column) and CD11b (right column). Both stainings are colored in green, blue color represents staining of nuclei with DAPI. The images were obtained using LSM 710 confocal microscope.

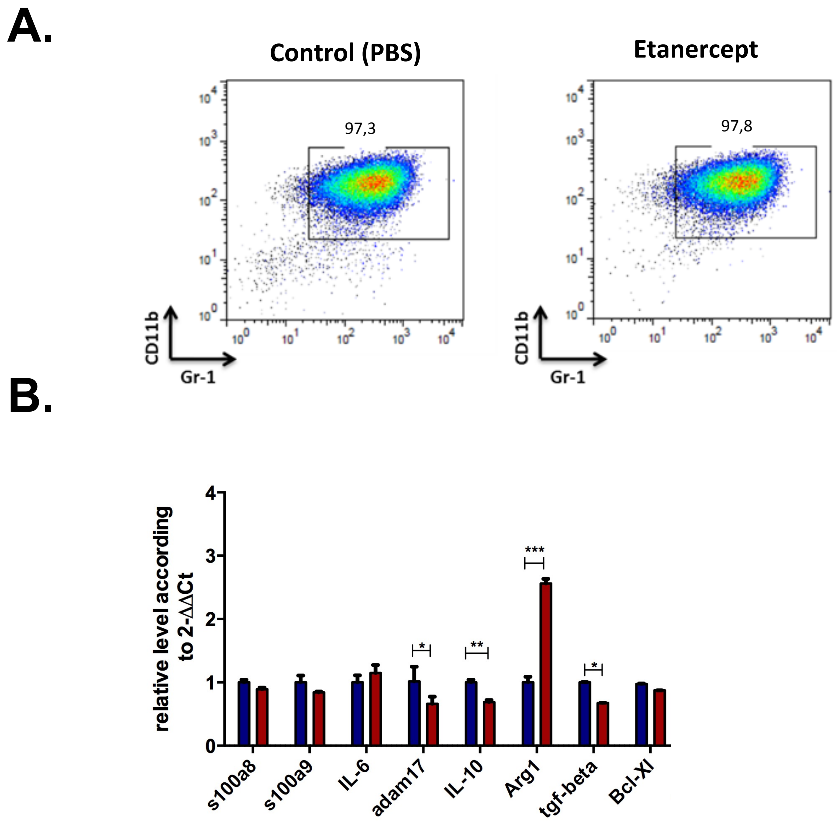

Supplementary figure 4. Systemic TNF ablation affects gene expression in splenic MDSC of tumor-bearing mice. A) CD11b+Gr-1+ cells were purified from the spleens of tumor-bearing mice using MDSC isolation kit according to manufacturer's protocols (Miltenyi Biotec, Germany). Flow cytometry analysis of purified splenic MDSC after magnetic separation. B) Gene expression profile of purified splenic MDSC of tumor-bearing mice treated with Etanercept (red) or PBS (blue). Each bar represents relative expression level according to $2^{-\Delta\Delta Ct}$ and normalized to expression level in MDSC from PBS-treated mice ±SD. Data is representative of two independent experiments. *$p < 0.05$, **$p < 0.01$, ***$p < 0.001$.
